# Supplementary material for: Optogenetic control of horizontally acquired genes prevent stuck fermentations in yeast
Source: Microbiol Spectr. 2025 Jan 8;13(2):e01794-24. doi: 10.1128/spectrum.01794-24 (PMC11792454; doi:10.1128/spectrum.01794-24)
Supplement: Supplemental figures and tables — Fig. S1 to S16; Tables S1 to S4. [file spectrum.01794-24-s0001.pdf]

Supplementary Materials

Optogenetic control of horizontally acquired genes prevent stuck fermentations in yeast

David Figueroa[1,2], Diego Ruiz[1,2], Eduardo I. Kessi-Pérez[3,4], Matteo De Chiara[5], Nicolò Tellini[5], Claudio Martínez[3,4], Gianni Liti[5], Amparo Querol[6], José M. Guillamón[6], Francisco Salinas[1,2*]

[1]Laboratorio de Genómica Funcional, Instituto de Bioquímica y Microbiología, Facultad de Ciencias, Universidad Austral de Chile, Valdivia, Chile.
[2]ANID–Millennium Science Initiative–Millennium Institute for Integrative Biology (iBio), Santiago, Chile.
[3]Centro de Estudios en Ciencia y Tecnología de los Alimentos (CECTA), Universidad de Santiago de Chile, Santiago, Chile.
[4]Departamento de Ciencia y Tecnología de los Alimentos, Facultad Tecnológica, Universidad de Santiago de Chile, Santiago, Chile.
[5]Université Côte d'Azur, CNRS, INSERM, IRCAN, Nice, France.
[6]Departamento de Biotecnología de los Alimentos, Instituto de Agroquímica y Tecnología de los Alimentos – Consejo Superior de Investigaciones Científicas (CSIC), Valencia, Spain

* Corresponding author email: francisco.salinas@uach.cl

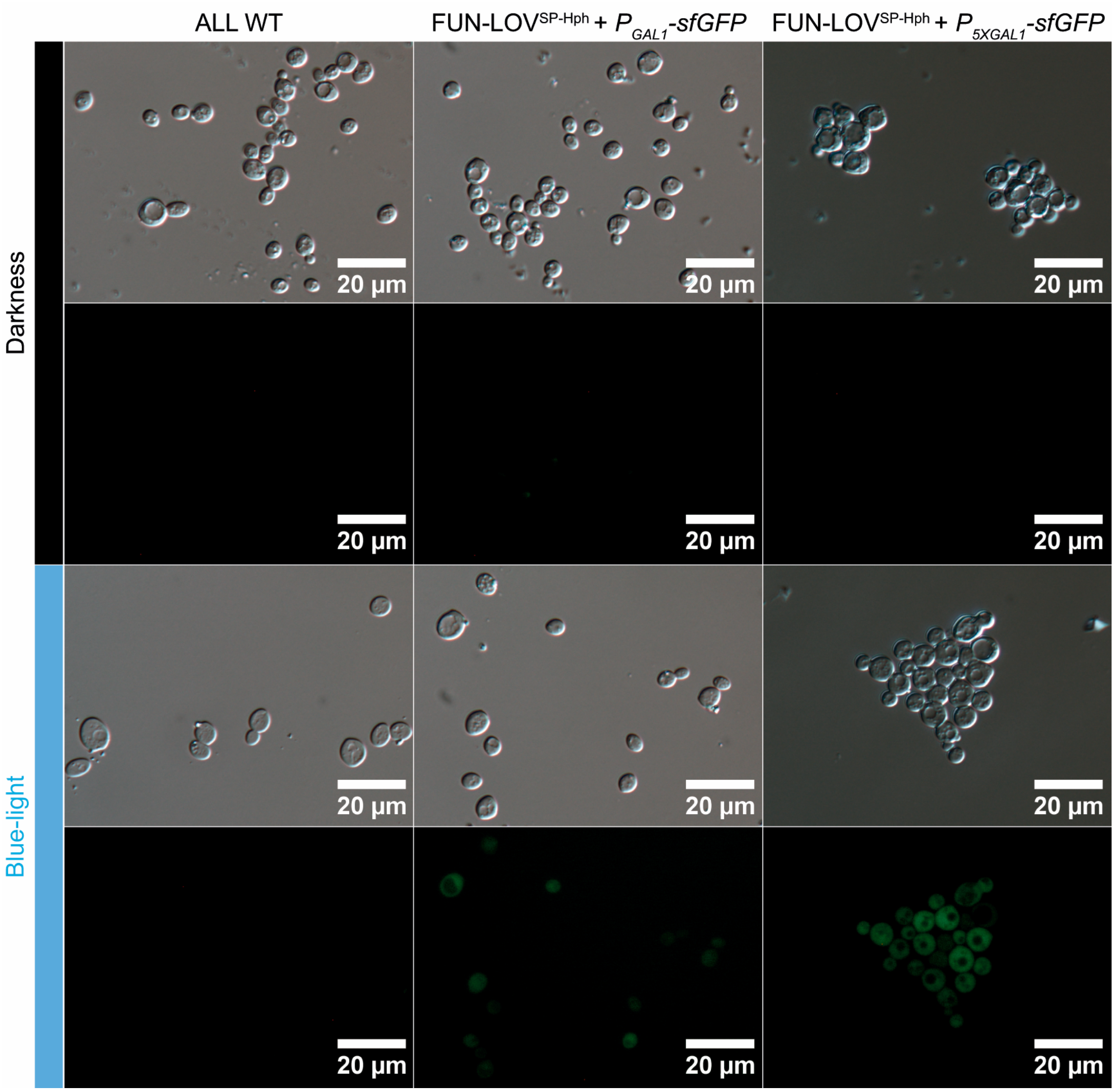

Figure S1. Fluorescence microscopy for strains expressing *sfGFP* under the control of FUN-LOV^{SP-Hph}. *sfGFP* was expressed under the control of the FUN-LOV^{SP-Hph} variant using either the P_{GAL1} or P_{5XGAL1} promoter. Fluorescence was evaluated in constant darkness (black bar, repression) or constant blue-light (blue bar, overexpression) conditions. Bright-field and fluorescence microscopy images are shown for each illumination condition. The scale bar represents 20 µm.

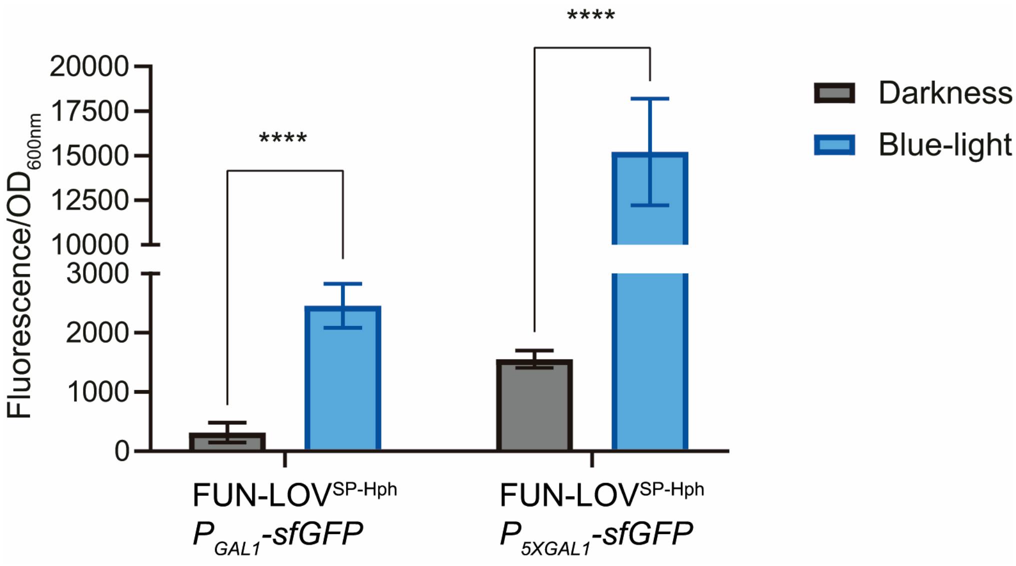

Figure S2. Fluorescence measurements in yeast strains expressing *sfGFP* under the control of the FUN-LOV$^{SP\text{-}Hph}$ variant. *sfGFP* was expressed under the control of either the P_{GAL1} or P_{5XGAL1} promoter, which are recognized by FUN-LOV$^{SP\text{-}Hph}$. Fluorescence of the yeast cultures was evaluated in constant darkness (black bars) and constant blue-light (blue bars) conditions. The final fluorescence of the yeast cultures was normalized by OD_{600nm}. The average of six biological replicates with the standard deviation represented as error bars is shown. The asterisks represent a statistically significant difference between constant darkness and constant blue-light conditions (*t*-test, **** = $p < 0.0001$).

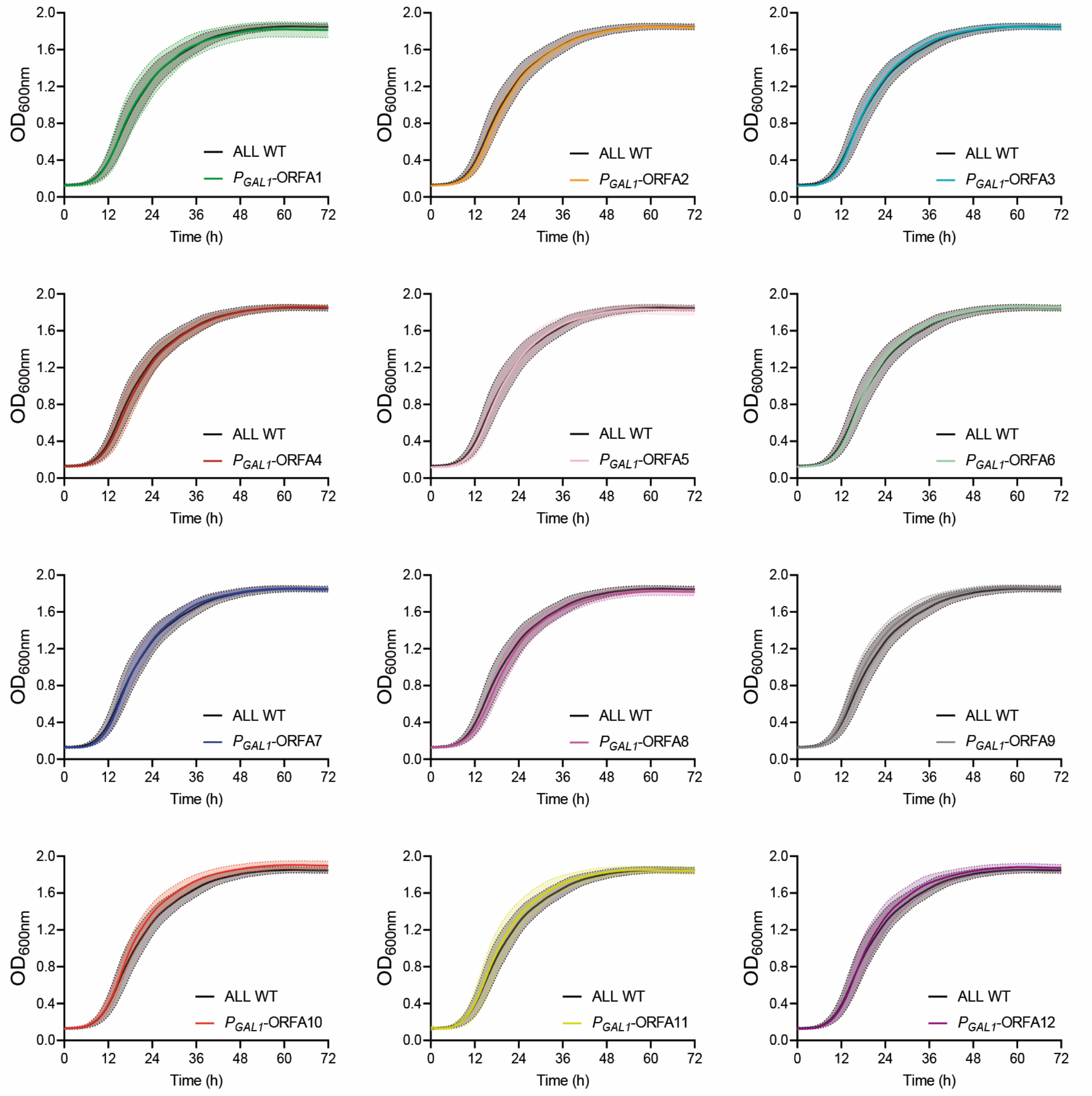

Figure S3. Raw data for growth curves in SM300 and constant darkness conditions. The panels show the growth kinetics measured as Optical Density OD at 600 nm (OD_{600nm}) for the 'ALL' wild type strain and derived strains carrying the FUN-LOV$^{SP\text{-}Hph}$ variant controlling different ORFs within region A. The *GAL1* promoter (P_{GAL1}) is recognized by the FUN-LOV$^{SP\text{-}Hph}$ variant. In all panels, the average of six biological replicates with the standard deviation represented as a color shaded region is shown.

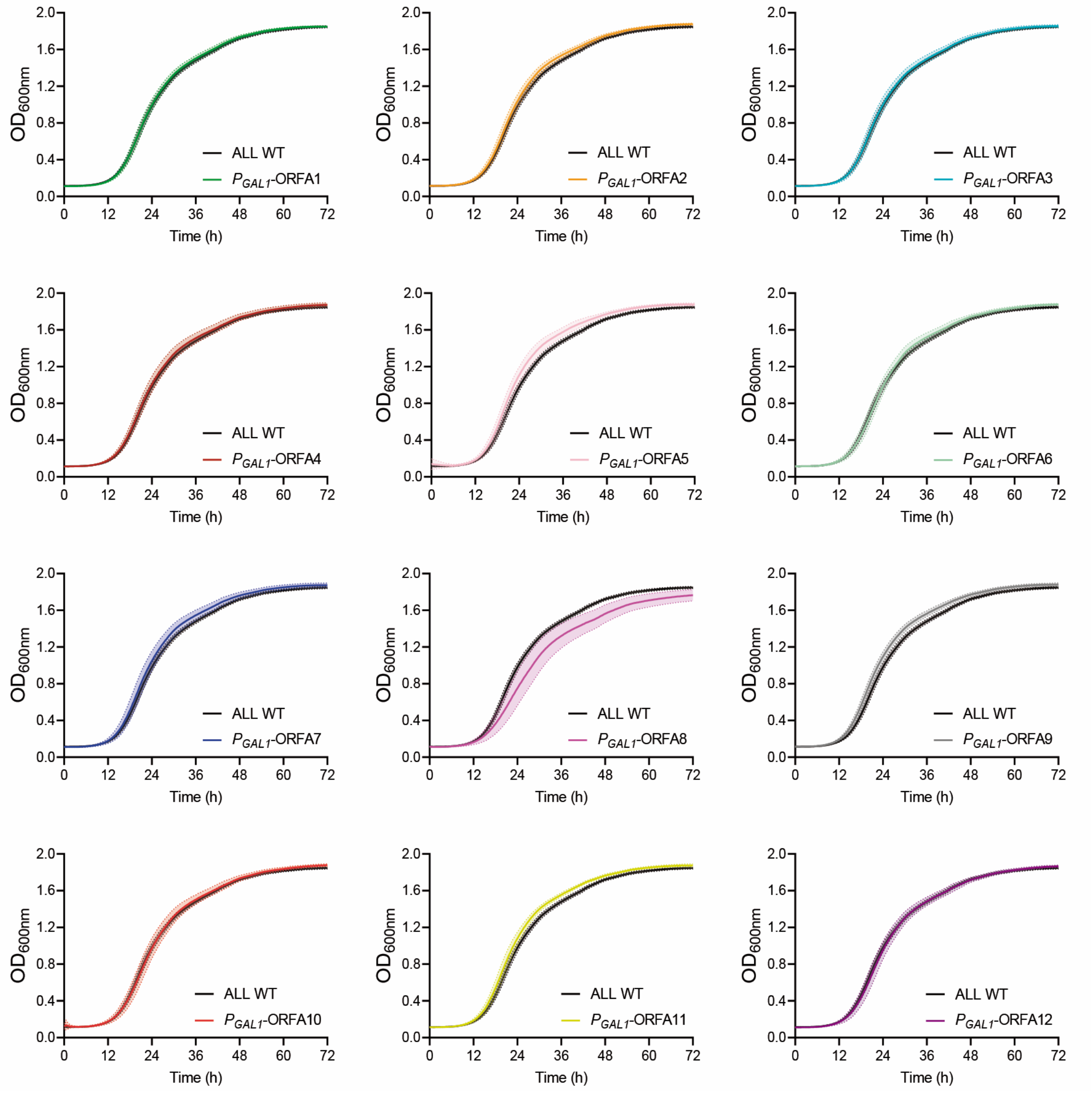

Figure S4. Raw data for growth curves in SM300 and constant blue-light conditions. The panels show the growth kinetics measured as Optical Density OD at 600 nm (OD_{600nm}) for the 'ALL' wild type strain and derived strains carrying the FUN-LOV$^{SP\text{-}Hph}$ variant controlling different ORFs within region A. The *GAL1* promoter (P_{GAL1}) is recognized by the FUN-LOV$^{SP\text{-}Hph}$ variant. In all panels, the average of six biological replicates with the standard deviation represented as a color shaded region is shown.

.

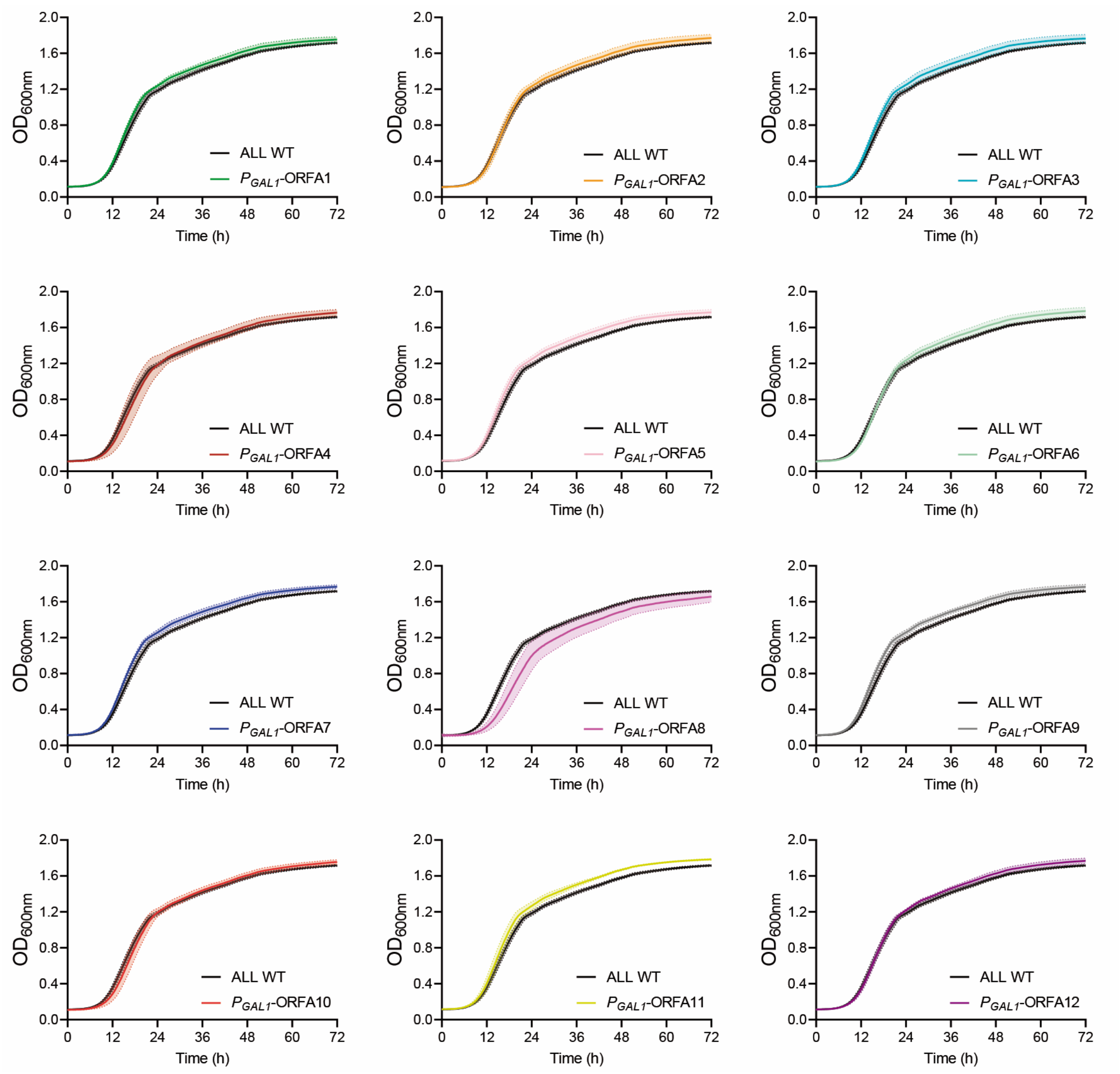

Figure S5. Raw data for growth curves in SM140 and constant darkness conditions. The panels show the growth kinetics measured as Optical Density OD at 600 nm (OD_{600nm}) for the 'ALL' wild type strain and derived strains carrying the FUN-LOV$^{SP\text{-}Hph}$ variant controlling different ORFs within region A. The *GAL1* promoter (P_{GAL1}) is recognized by the FUN-LOV$^{SP\text{-}Hph}$ variant. In all panels, the average of six biological replicates with the standard deviation represented as a color shaded region is shown.

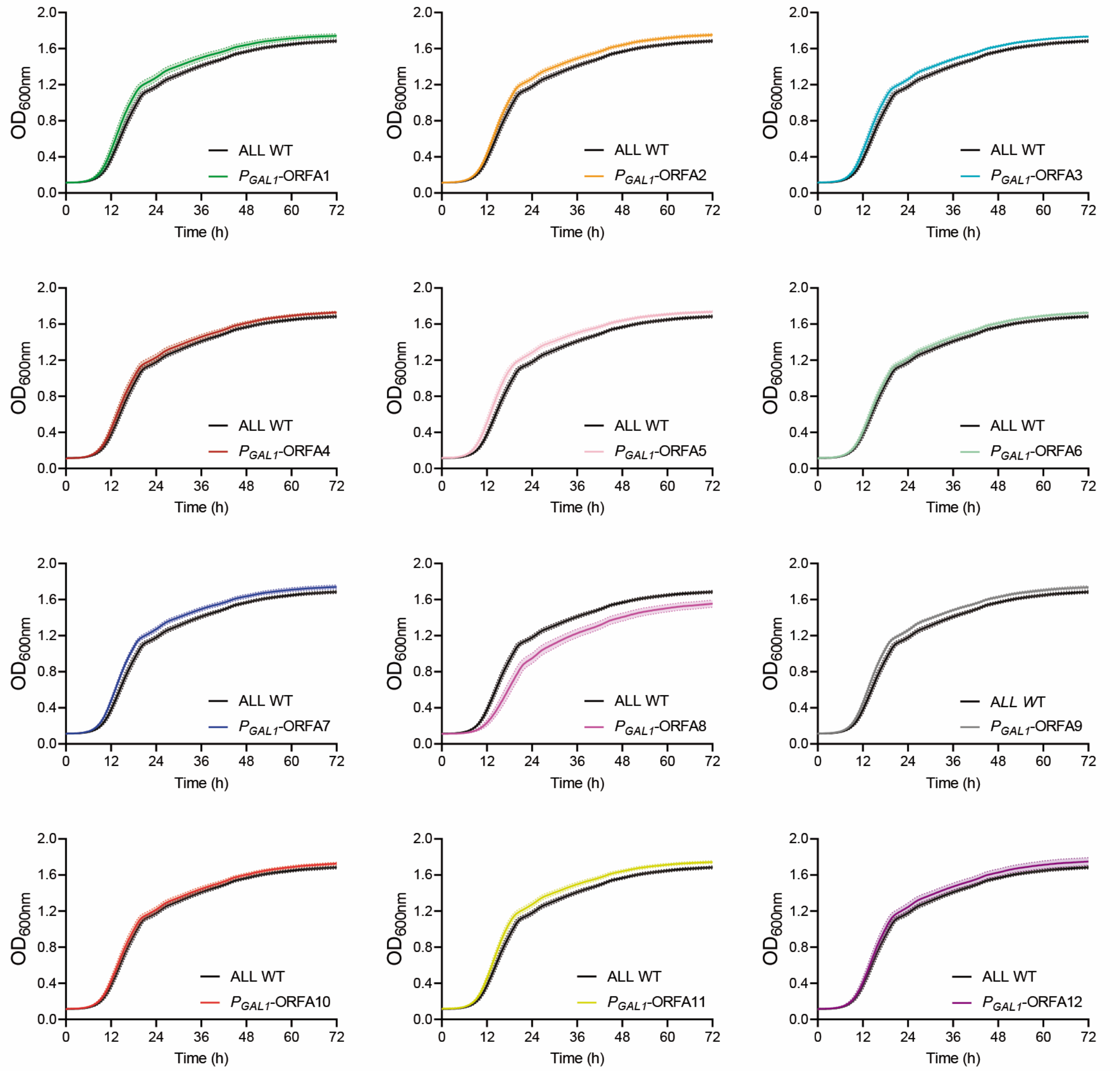

Figure S6. Raw data for growth curves in SM140 and constant blue-light conditions. The panels show the growth kinetics measured as Optical Density OD at 600 nm (OD_{600nm}) for the 'ALL' wild type strain and derived strains carrying the FUN-LOV$^{SP\text{-}Hph}$ variant controlling different ORFs within region A. The *GAL1* promoter (P_{GAL1}) is recognized by the FUN-LOV$^{SP\text{-}Hph}$ variant. In all panels, the average of six biological replicates with the standard deviation represented as a color shaded region is shown.

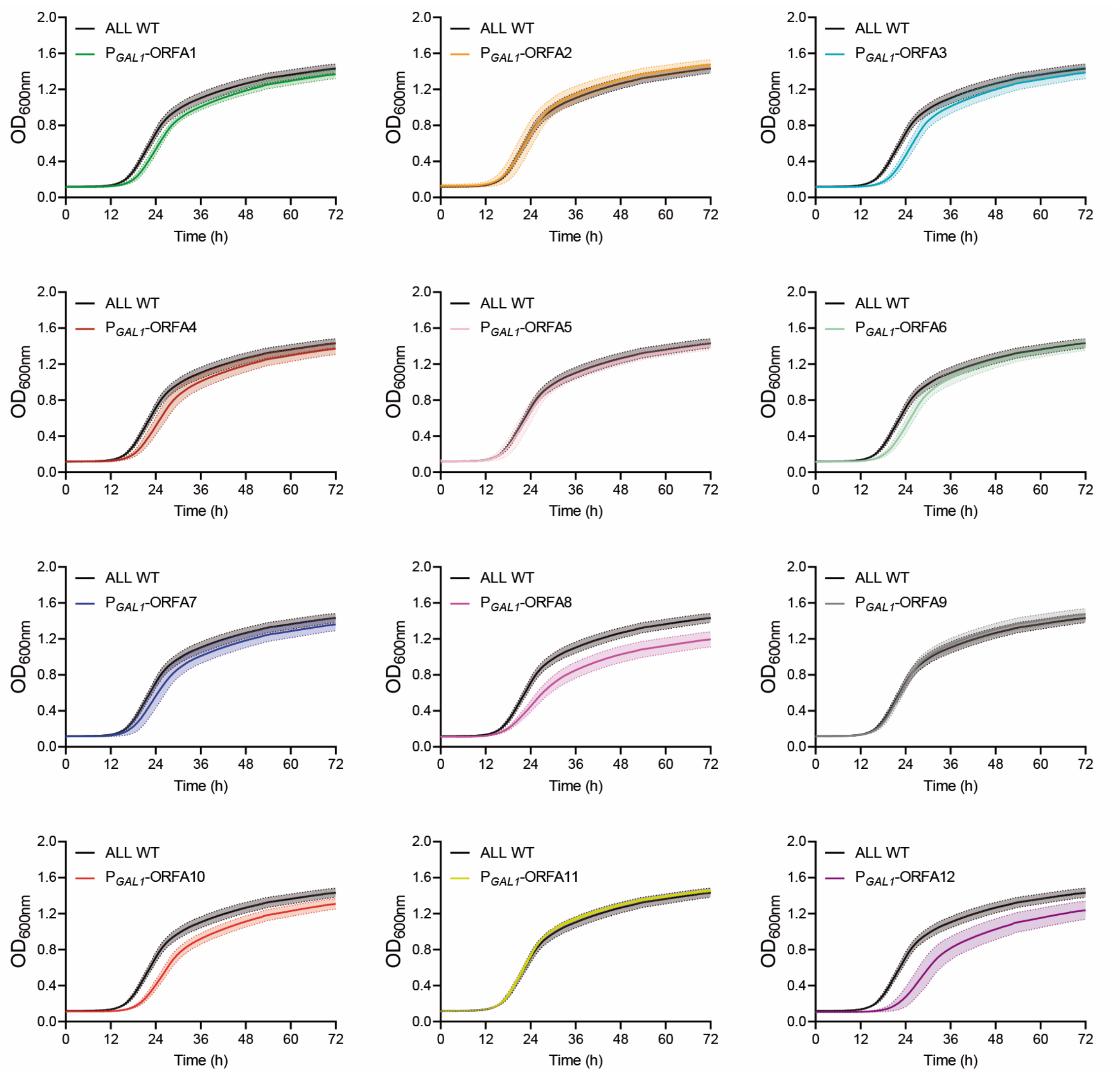

Figure S7. Raw data for growth curves in SM60 and constant darkness conditions. The panels show the growth kinetics measured as Optical Density OD at 600 nm (OD_{600nm}) for the 'ALL' wild type strain and derived strains carrying the FUN-LOV$^{SP\text{-}Hph}$ variant controlling different ORFs within region A. The *GAL1* promoter (P_{GAL1}) is recognized by the FUN-LOV$^{SP\text{-}Hph}$ variant. In all panels, the average of six biological replicates with the standard deviation represented as a color shaded region is shown.

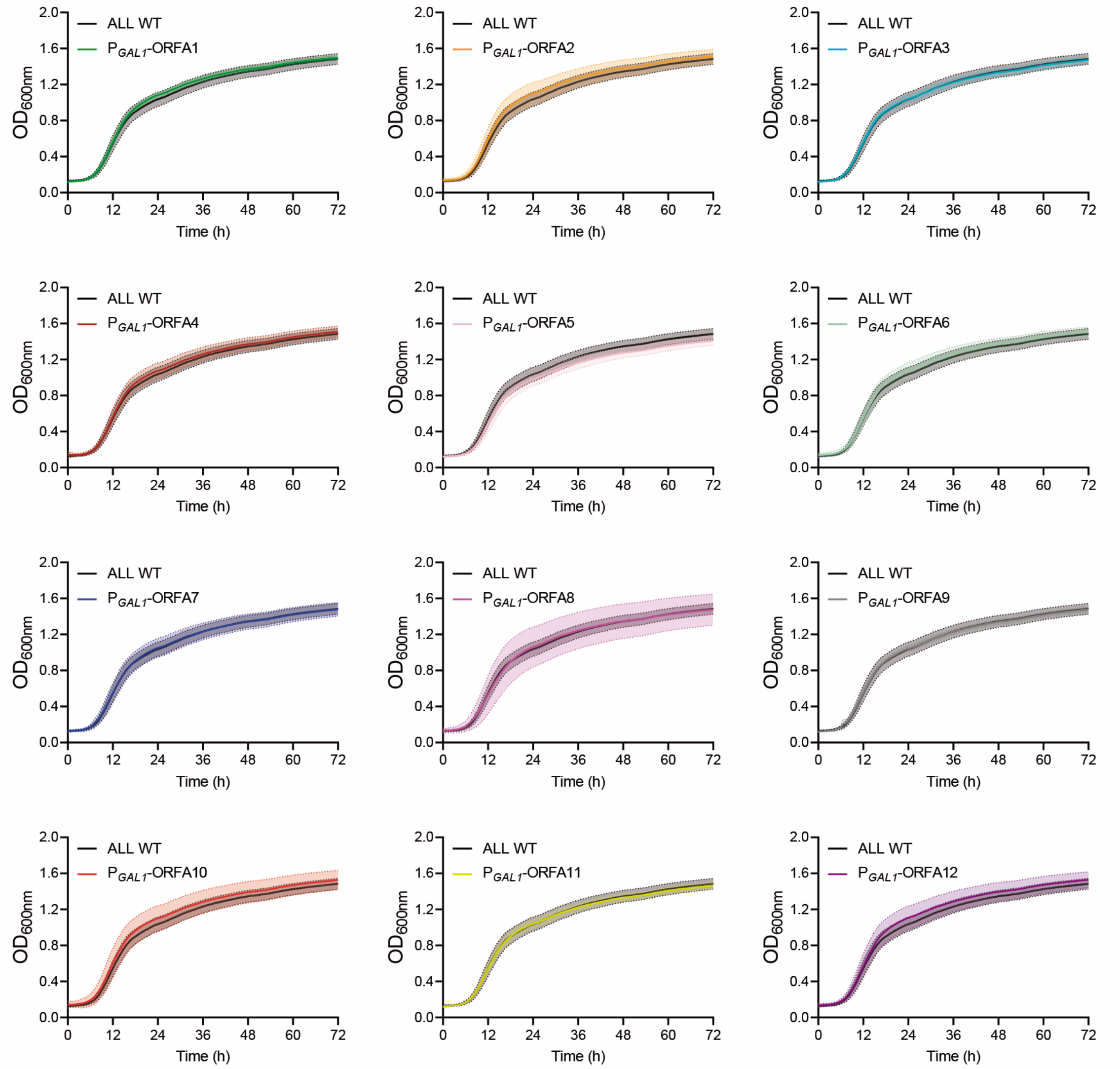

Figure S8. Raw data for growth curves in SM60 and constant blue-light conditions. The panels show the growth kinetics measured as Optical Density OD at 600 nm (OD_{600nm}) for the 'ALL' wild type strain and derived strains carrying the FUN-LOV$^{SP\text{-}Hph}$ variant controlling different ORFs within region A. The *GAL1* promoter (P_{GAL1}) is recognized by the FUN-LOV$^{SP\text{-}Hph}$ variant. In all panels, the average of six biological replicates with the standard deviation represented as a color shaded region is shown.

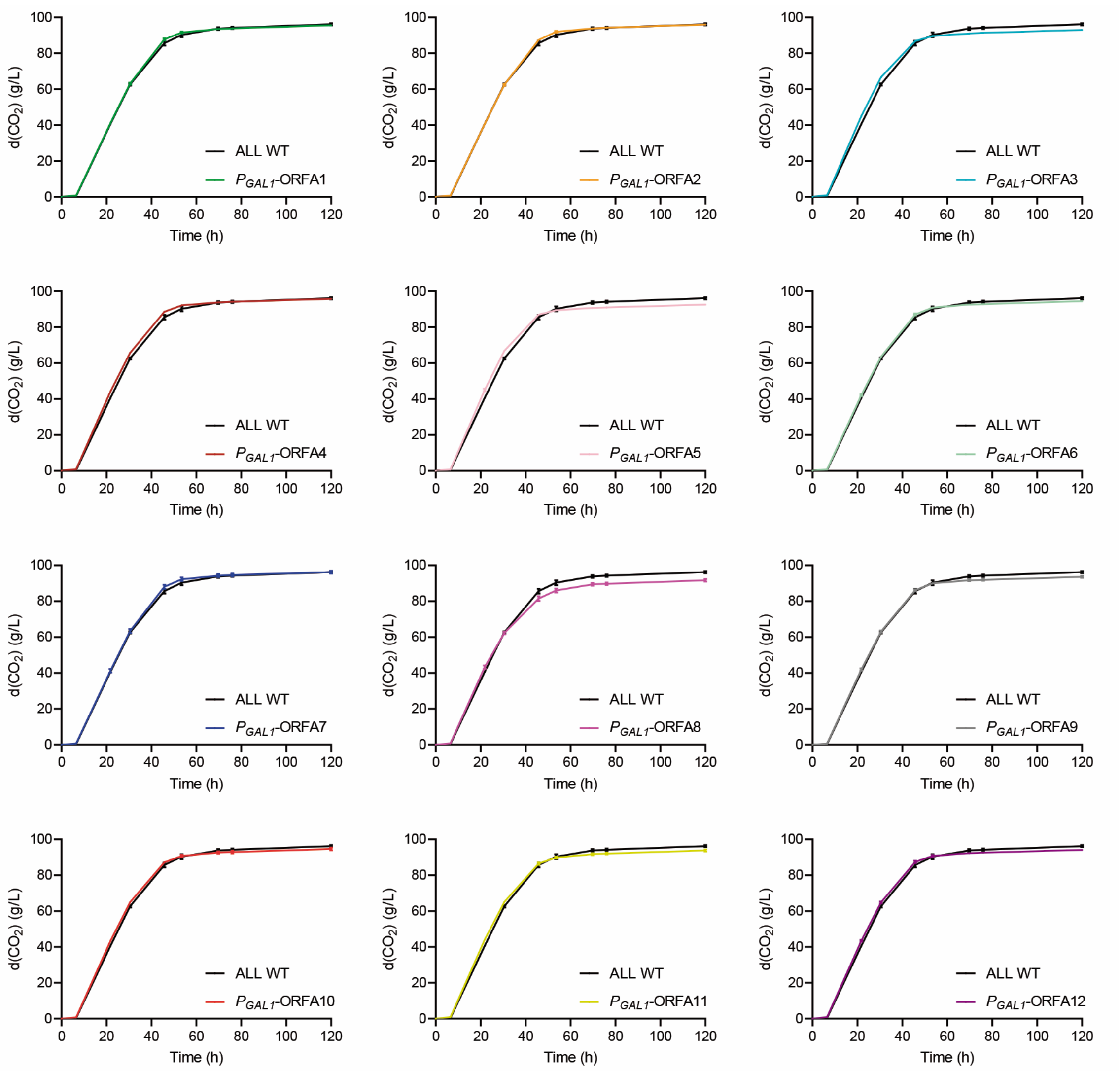

Figure S9. Raw data for fermentations in SM300 and constant darkness conditions. The panels show the fermentation kinetics measured as CO_2 loss for the 'ALL' wild type strain and derived versions carrying the FUN-LOV$^{SP\text{-}Hph}$ variant controlling different ORFs within region A. The *GAL1* promoter (P_{GAL1}) is recognized by the FUN-LOV$^{SP\text{-}Hph}$ variant. In all panels, the average of three biological replicates with the standard deviation represented as error bars is shown.

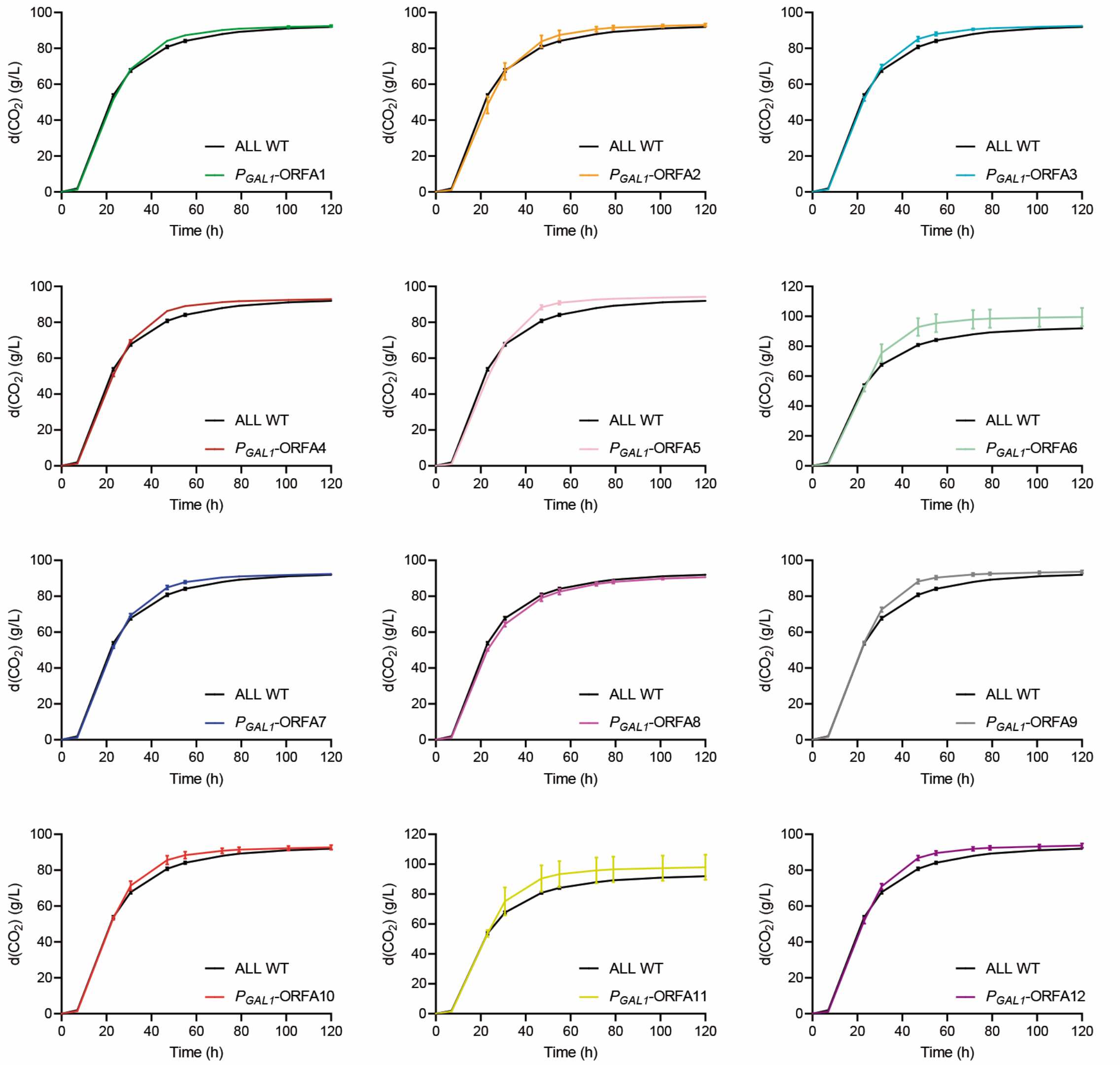

Figure S10. Raw data for fermentations in SM300 and constant blue-light conditions. The panels show the fermentation kinetics measured as CO_2 loss for the 'ALL' wild type strain and derived versions carrying the FUN-LOV$^{SP\text{-}Hph}$ variant controlling different ORFs within region A. The *GAL1* promoter (P_{GAL1}) is recognized by the FUN-LOV$^{SP\text{-}Hph}$ variant. In all panels, the average of three biological replicates with the standard deviation represented as error bars is shown.

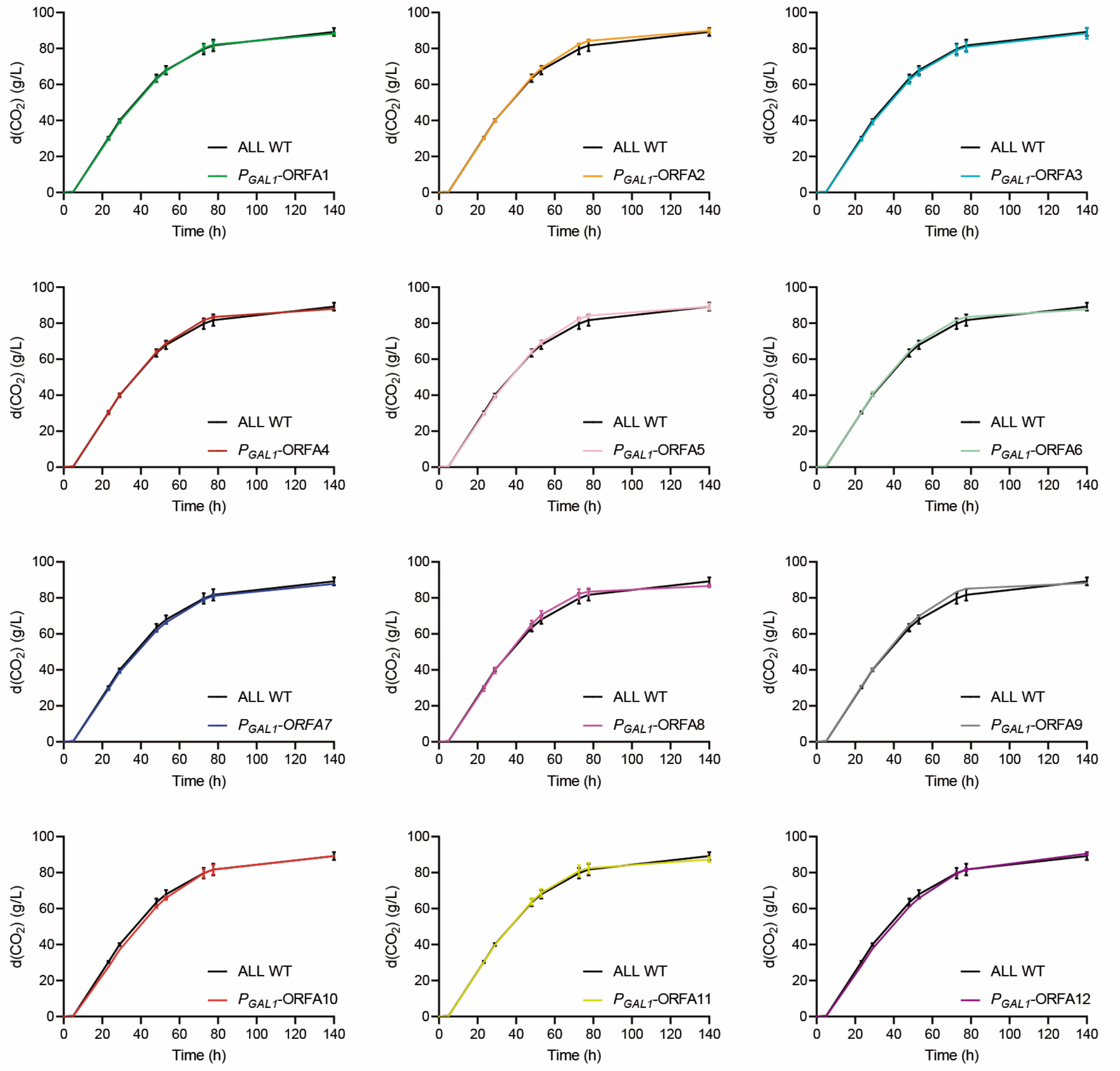

Figure S11. Raw data for fermentations in SM140 and constant darkness conditions. The panels show the fermentation kinetics measured as CO_2 loss for the 'ALL' wild type strain and derived versions carrying the FUN-LOV$^{SP\text{-}Hph}$ variant controlling different ORFs within region A. The *GAL1* promoter (P_{GAL1}) is recognized by the FUN-LOV$^{SP\text{-}Hph}$ variant. In all panels, the average of three biological replicates with the standard deviation represented as error bars is shown.

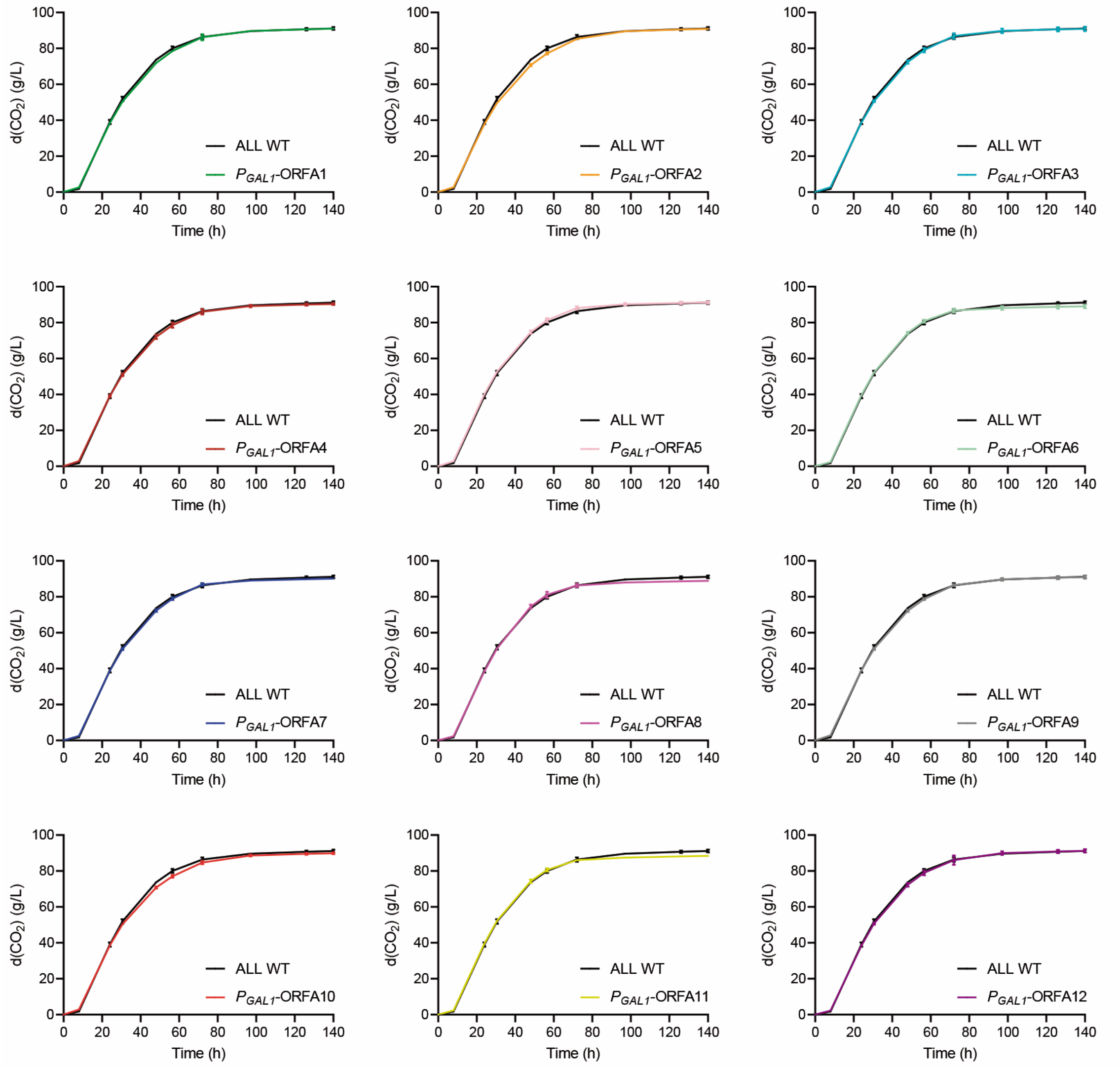

Figure S12. Raw data for fermentations in SM140 and constant blue-light conditions. The panels show the fermentation kinetics measured as CO_2 loss for the 'ALL' wild type strain and derived versions carrying the FUN-LOV$^{SP\text{-}Hph}$ variant controlling different ORFs within region A. The *GAL1* promoter (P_{GAL1}) is recognized by the FUN-LOV$^{SP\text{-}Hph}$ variant. In all panels, the average of three biological replicates with the standard deviation represented as error bars is shown.

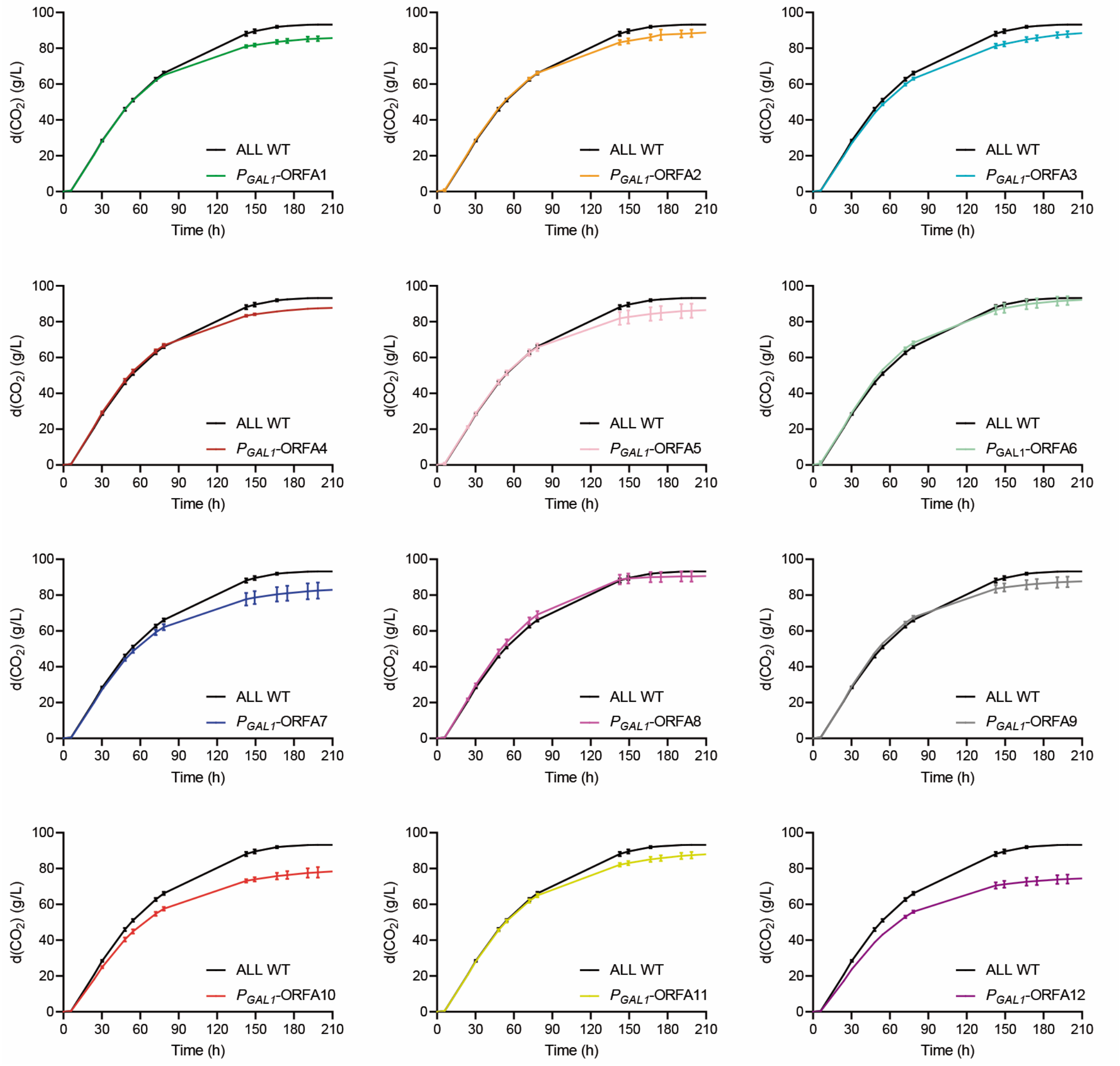

Figure S13. Raw data for fermentations in SM60 and constant darkness conditions. The panels show the fermentation kinetics measured as CO_2 loss for the 'ALL' wild type strain and derived versions carrying the FUN-LOV$^{SP\text{-}Hph}$ variant controlling different ORFs within region A. The *GAL1* promoter (P_{GAL1}) is recognized by the FUN-LOV$^{SP\text{-}Hph}$ variant. In all panels, the average of three biological replicates with the standard deviation represented as error bars is shown.

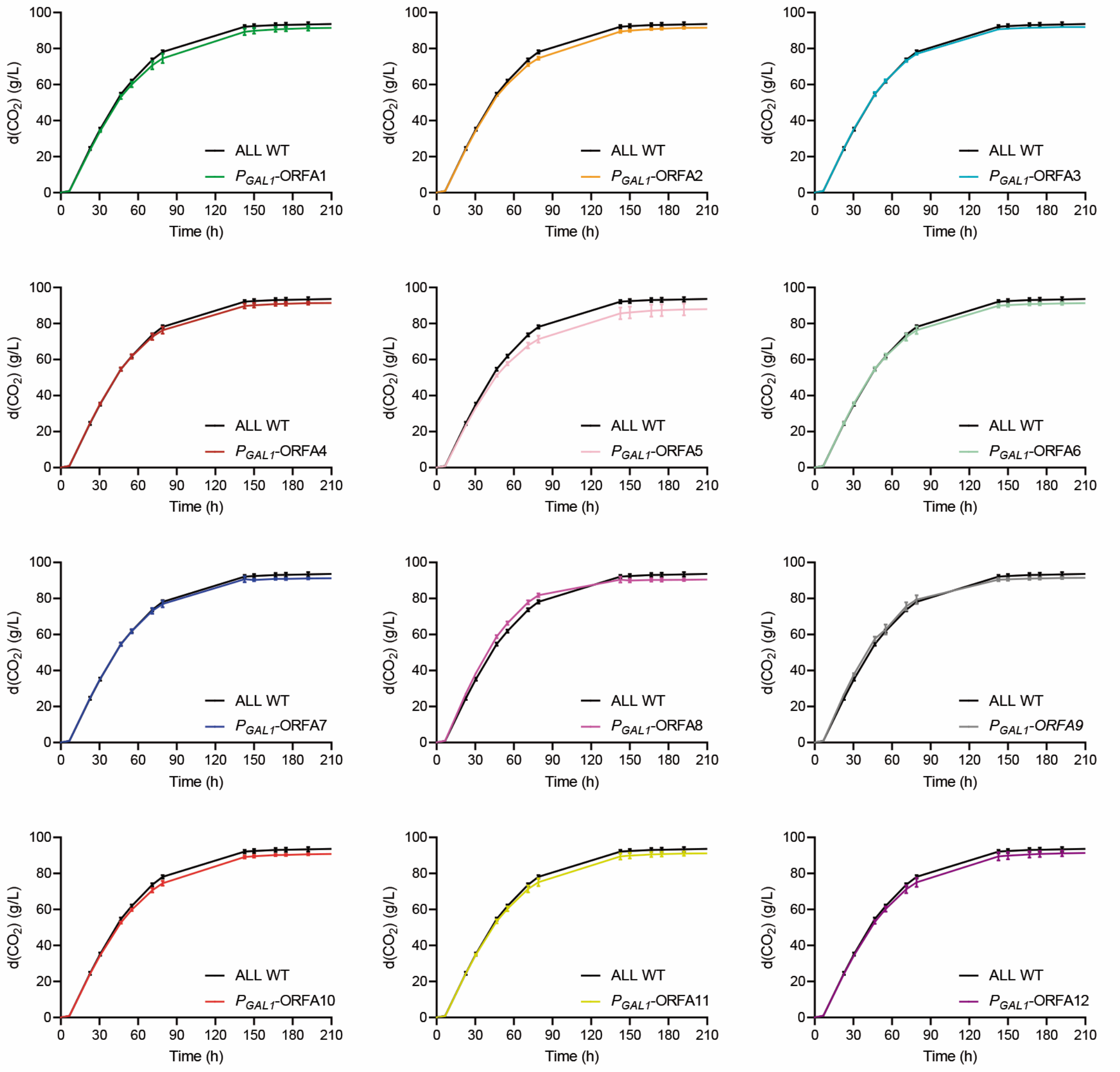

Figure S14. Raw data for fermentations in SM60 and constant blue-light conditions. The panels show the fermentation kinetics measured as CO_2 loss for the 'ALL' wild type strain and the derived versions carrying the FUN-LOV$^{SP\text{-}Hph}$ variant controlling different ORFs within region A. The *GAL1* promoter (P_{GAL1}) is recognized by the FUN-LOV$^{SP\text{-}Hph}$ variant. In all panels, the average of three biological replicates with the standard deviation represented as error bars is shown.

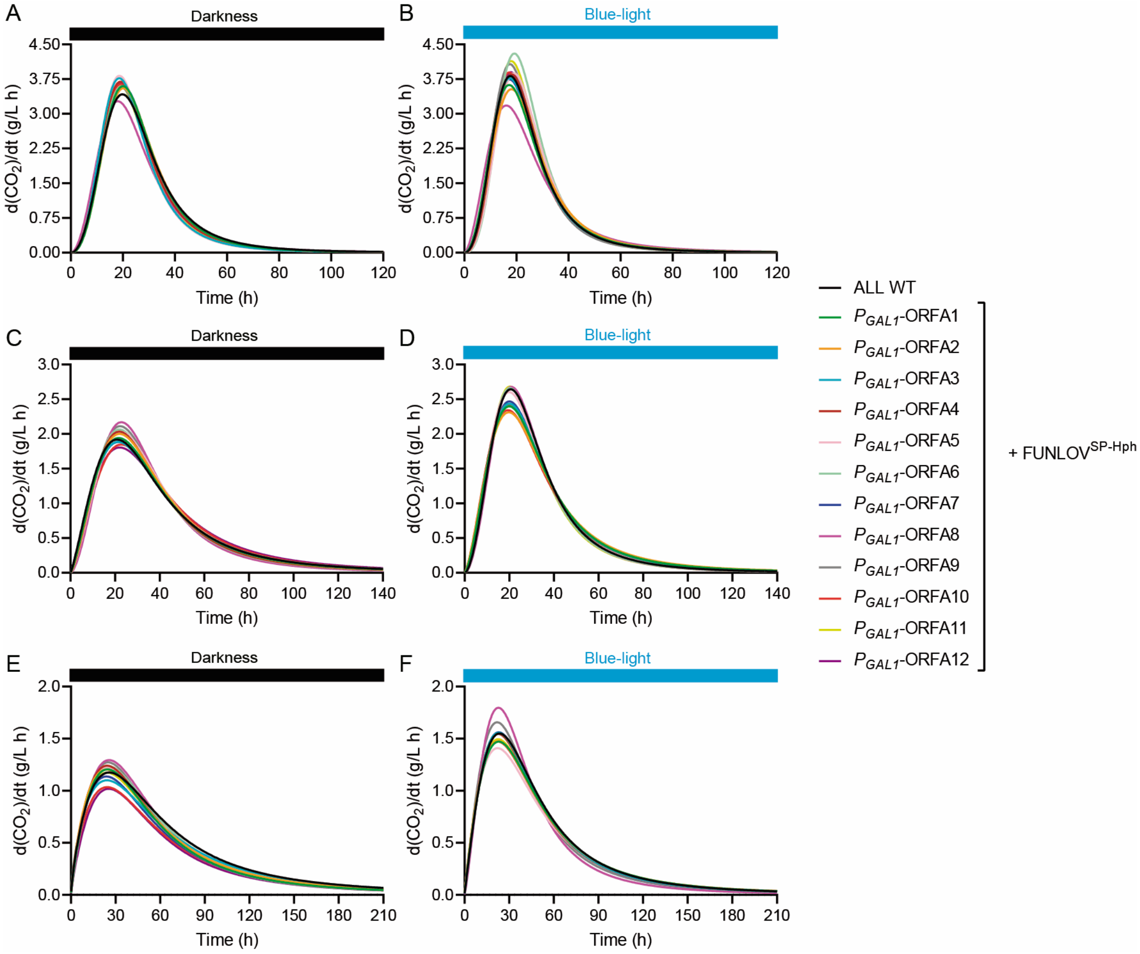

Figure S15. Maximal CO_2 production rate (V_{max}) derived from fermentation kinetics. The 'ALL' wine yeast strain and derived strains carrying the FUN-LOV$^{SP\text{-}Hph}$ variant controlling the expression of different ORFs within region A were subjected to fermentations in SM300 (panels A and B), SM140 (panels C and D), and SM60 (panels E and F). Fermentations were performed in constant darkness (panels A, C, and E) and constant blue-light (panels B, D, and F) conditions. The CO_2 release curves were fitted to a sigmoid non-linear regression, and the first derivate was calculated to obtain the maximal CO_2 production rate (V_{max}, peak of the curves). The average of three biological replicates is shown.

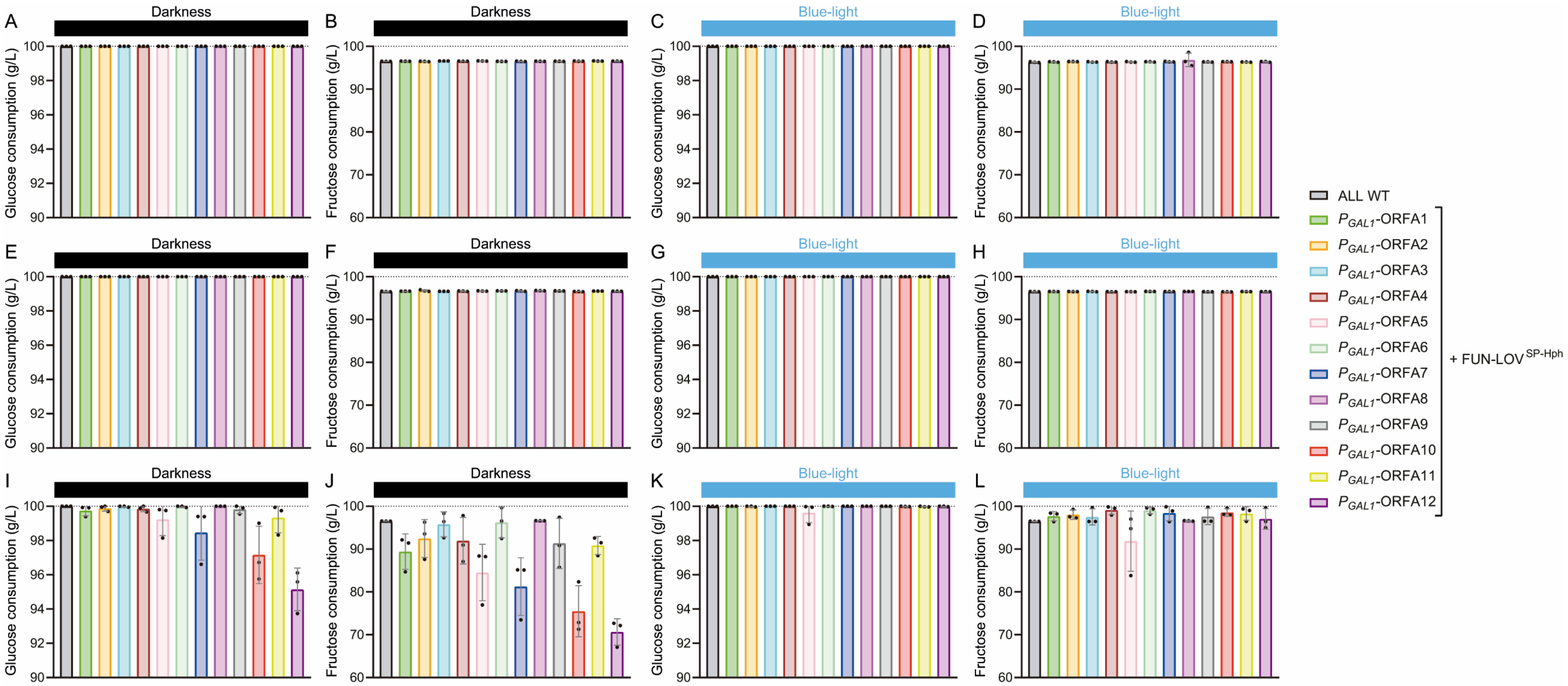

Figure S16. Sugar consumption at the end of the fermentations. Fermentations were performed in SM300 (panels A, B, C, and D), SM140 (panels E, F, G, and H), and SM60 (panels I, J, K, and L) for the 'ALL' wine yeast strain and derived strains carrying the FUN-LOV^{SP-Hph} variant controlling the expression of different ORFs within region A. Fermentations were performed in constant darkness (panels A, B, E, F, I, and J) and constant blue-light (panels C, D, G, H, K, and L) conditions. The fermentation medium was supplemented with 100 g/L of glucose and 100 g/L of fructose (dashed lines) and consumption were determined by HPLC. In all panels, the average of three biological replicates with the standard deviation represented as error bars is shown.

Table S1. Putative functions and orthologous genes for horizontally acquired genes of Region A in the 'ALL' wine yeast strain.

**Order in the HGT region**	**Hypothetical function**	**EC1118 or S288C ortholog**
A1	Hypothetical protein, putative monooxygenase	No ortholog found
A2	Enoyl reductase-like probably dehydrogenase (D-arabinose 1-dehydrogenase)	*YCR102C/YNL134C/ YLR460C*
A3	Sugar (or other) transporter, probably hexose transporter, or plasma membrane high glucose sensor	*RGT2/HXT10/HXT5/ STL1/SNF3*
A4	Aldehyde reductase	*YDR541C/GRE2/ ARI1*
A5	Galactose mutarotase-like, probably UDP-glucose-4-epimerase	*GAL10*
A6	Fungal transcription factor, probably involved in regulating lysine biosynthesis	*LYS14*
A7	Arginase	*CAR1*
A8	Member of the multidrug and toxic compound extrusion (MATE) family, probably, Na+ -driven multidrug efflux pump	*ERC1/YDR338C*
A9	Contains a solute binding domain of nucleobase-cation-symport-1 (NCS1) transporter NRT1-like probably transporter of thiamine or related compound	*THI72/THI7*
A10	Methyltransferase	*COQ5*
A11	FAD/FMN-containing dehydrogenase, probably 2-hydroxyglutarate transhydrogenase and minor D-lactate dehydrogenase	*DLD3/DLD2*
A12	2-Nitropropane dioxygenase or nitronate monooxygenase	*YJR149W*

Table S2. Yeast strains used and generated in this work.

Strain	Genotype	Source
ALL WT	-	Peter et al., 2018
P_{GAL1}-*Luc* + FUN-LOV$^{SP\text{-}Hph}$	ALL; *gal3*Δ::KanMxRV P_{GAL1}-*Luc*; *ho*Δ::FUN-LOV$^{SP\text{-}Hph}$	This work
P_{5XGAL1}-*Luc* + FUN-LOV$^{SP\text{-}Hph}$	ALL; *gal3*Δ::KanMxRV P_{5XGAL1}-*Luc*; *ho*Δ::FUN-LOV$^{SP\text{-}Hph}$	This work
P_{GAL1}-*sfGFP* + FUN-LOV$^{SP\text{-}Hph}$	ALL; *gal3*Δ::KanMxRV P_{GAL1}-*sfGFP*; *ho*Δ::FUN-LOV$^{SP\text{-}Hph}$	This work
P_{5XGAL1}-*sfGFP* + FUN-LOV$^{SP\text{-}Hph}$	ALL; *gal3*Δ::KanMxRV P_{5XGAL1}-*sfGFP*; *ho*Δ::FUN-LOV$^{SP\text{-}Hph}$	This work
P_{GAL1}-ORFA1 + FUN-LOV$^{SP\text{-}Hph}$	ALL; P_{ORFA1}Δ::KanMxRV-P_{GAL1}; *ho*Δ::FUN-LOV$^{SP\text{-}Hph}$	This work
P_{GAL1}-ORFA2 + FUN-LOV$^{SP\text{-}Hph}$	ALL; P_{ORFA2}Δ::KanMxRV-P_{GAL1}; *ho*Δ::FUN-LOV$^{SP\text{-}Hph}$	This work
P_{GAL1}-ORFA3 + FUN-LOV$^{SP\text{-}Hph}$	ALL; P_{ORFA3}Δ::KanMxRV-P_{GAL1}; *ho*Δ::FUN-LOV$^{SP\text{-}Hph}$	This work
P_{GAL1}-ORFA4 + FUN-LOV$^{SP\text{-}Hph}$	ALL; P_{ORFA4}Δ::KanMxRV-P_{GAL1}; *ho*Δ::FUN-LOV$^{SP\text{-}Hph}$	This work
P_{GAL1}-ORFA5 + FUN-LOV$^{SP\text{-}Hph}$	ALL; P_{ORFA5}Δ::KanMxRV-P_{GAL1}; *ho*Δ::FUN-LOV$^{SP\text{-}Hph}$	This work
P_{GAL1}-ORFA6 + FUN-LOV$^{SP\text{-}Hph}$	ALL; P_{ORFA6}Δ::KanMxRV-P_{GAL1}; *ho*Δ::FUN-LOV$^{SP\text{-}Hph}$	This work
P_{GAL1}-ORFA7 + FUN-LOV$^{SP\text{-}Hph}$	ALL; P_{ORFA7}Δ::KanMxRV-P_{GAL1}; *ho*Δ::FUN-LOV$^{SP\text{-}Hph}$	This work
P_{GAL1}-ORFA8 + FUN-LOV$^{SP\text{-}Hph}$	ALL; P_{ORFA8}Δ::KanMxRV-P_{GAL1}; *ho*Δ::FUN-LOV$^{SP\text{-}Hph}$	This work
P_{GAL1}-ORFA9 + FUN-LOV$^{SP\text{-}Hph}$	ALL; P_{ORFA9}Δ::KanMxRV-P_{GAL1}; *ho*Δ::FUN-LOV$^{SP\text{-}Hph}$	This work
P_{GAL1}-ORFA10 + FUN-LOV$^{SP\text{-}Hph}$	ALL; P_{ORFA10}Δ::KanMxRV-P_{GAL1}; *ho*Δ::FUN-LOV$^{SP\text{-}Hph}$	This work
P_{GAL1}-ORFA11 + FUN-LOV$^{SP\text{-}Hph}$	ALL; P_{ORFA11}Δ::KanMxRV-P_{GAL1}; *ho*Δ::FUN-LOV$^{SP\text{-}Hph}$	This work
P_{GAL1}-ORFA12 + FUN-LOV$^{SP\text{-}Hph}$	ALL; P_{ORFA12}Δ::KanMxRV-P_{GAL1}; *ho*Δ::FUN-LOV$^{SP\text{-}Hph}$	This work
P_{5XGAL1}-ORFA6 + FUN-LOV$^{SP\text{-}Hph}$	ALL; P_{ORFA6}Δ::KanMxRV-P_{5XGAL1}; *ho*Δ::FUN-LOV$^{SP\text{-}Hph}$	This work
P_{5XGAL1}-ORFA8 + FUN-LOV$^{SP\text{-}Hph}$	ALL; P_{ORFA8}Δ::KanMxRV-P_{5XGAL1}; *ho*Δ::FUN-LOV$^{SP\text{-}Hph}$	This work

Table S3. List of primers used and generated in this work.

Description	Type	Length (nt)	Sequence (5´-3´)
Swapping *GAL3* locus	Fw	70	AGGAGTGCAAAAAGAGAAAATAAAAGTAAAAAGGTAGG GCAACACATAGTATCGATGAATTCGAGCTCGT
Swapping *GAL3* locus	Rv	70	TATGAGTAAACTTTTAATATTTAAAGGTTGTTCCAAGAAGG TGTTTAGTGTGGATCCTTGCAAATTAAAG
GAL3 Upstream	Fw	20	ATGAAATCGCCATGCCAAGC
GAL3 Downstream	Rv	20	GTGCGGAGCCACTCTGACTC
K3: internal KanMx	Fw	21	CATCCTATGGAACTGCCTCGG
Internal *Luc*	Fw	20	ATCGTGGTGTGCTCTGAGAA
Assemble pRS316-KanMx	Fw	60	GGCCAGTGAATTGTAATACGACTCACTATAGGGCGAATTG ATCGATGAATTCGAGCTCGT
Assemble $P_{GAL1/5XGAL1}$-*sfGFP*	Fw	60	AGCTGTAATACGACTCACTATAGGGAATATTAAGCTTACCA TGCGTAAAGGCGAAGAGCT
Assemble $P_{GAL1/5XGAL1}$-*sfGFP*	Rv	60	TAGGGACGACACCAGTGAACAGCTCTTCGCCTTTACGCAT GGTAAGCTTAATATTCCCTA
Assemble *sfGFP*-$CYC1_{ter}$	Fw	60	AGCGGGCATCACGCATGGTATGGATGAACTGTACAAATGA TCATGTAATTAGTTATGTCA
Assemble *sfGFP*-$CYC1_{ter}$	Rv	60	GGGGAGGGCGTGAATGTAAGTGACATAACTAATTACATGA TCATTTGTACAGTTCATCCA
Assemble $CYC1_{ter}$-pRS316	Rv	60	CAAGCTCGGAATTAACCCTCACTAAAGGGAACAAAAGCT GTGGATCCTTGCAAATTAAAG
pRS316 Upstream	Fw	20	TTCGCTATTACGCCAGCTGG
pRS316 Downstream	Rv	20	TGCTTCCGGCTCCTATGTTG
Swapping *HO* locus	Fw	70	TCTAAATCCATATCCTCATAAGCAGCAATCAATTCTATCTAT ACTTTAAAATCGATGAATTCGAGCTCGT
Swapping *HO* locus	Rv	70	ATTAAATTTTACTTTTATTACATACAACTTTTTAAACTAATA TACACATTTGGATCCTTGCAAATTAAAG
HO Upstream	Fw	20	GAATTGTACTACCGCTGGGC
HO Downstream	Rv	22	TGGTTGAAACAAATCAGTGCCG
Swapping P_{ORFA1}	Fw	70	CCCAAACCATTATTTGCAGTTGATGCACGCTTCCCGTTCTC AGTTGTCATGGTAAGCTTAATATTCCCTA
Swapping P_{ORFA1}	Rv	70	GGTTCGCTATTTGAGTACGTGGACTTGGGTCTGCTTGAAA CGCTACGATGATCGATGAATTCGAGCTCGT
Swapping P_{ORFA1} confirmation	Rv	20	CCAGGTGCTGCTTCACGGGT
Swapping P_{ORFA2}	Fw	70	ACTCGGAGTATTTTATTTATTGTCTAGCCTTTAATTGCGGAT GCGCCTTCATCGATGAATTCGAGCTCGT
Swapping P_{ORFA2}	Rv	70	GACGATTCTCCTATGTAATGATGAAAATCAGGGTATGATCT TTTTAACATGGTAAGCTTAATATTCCCTA
Swapping P_{ORFA2} confirmation	Fw	20	CGCGGACTTGTCCTTGGGGT

Swapping P_{ORFA3}	Fw	70	ATACTAGATTTAGATTGTCTTTACATTAATAATCTAGATTCT TCAGTTTCATCGATGAATTCGAGCTCGT
Swapping P_{ORFA3}	Rv	70	ACTTCATCAATTGCGATGGCTTTTTCGTCGAGGTTTTTGGT TTCAGACATGGTAAGCTTAATATTCCCTA
Swapping P_{ORFA3} confirmation	Fw	20	AGACTGGTATGCGTCGTTGG
Swapping P_{ORFA4}	Fw	70	ATATGTTGAGCTATAAATCCATTTGCACCCGAAACCAACAC AGCAGACATGGTAAGCTTAATATTCCCTA
Swapping P_{ORFA4}	Rv	70	ACGCGCACCCTTACATTTCTCGAAGTTCTTCAAAGCTTCA GACAGGGCCAATCGATGAATTCGAGCTCGT
Swapping P_{ORFA4} confirmation	Rv	20	GGGGGCCAAACAGCGGATGT
Swapping P_{ORFA5}	Fw	70	CCAGTCACCAGCACCCATTTGCCTGATATTTTTCTTTCTGT CGTTCTCATGGTAAGCTTAATATTCCCTA
Swapping P_{ORFA5}	Rv	70	GGCACCAGTGTAATTGTGGTTGAGGAGGATTACACCTCCA AGACCTGCTCATCGATGAATTCGAGCTCGT
Swapping P_{ORFA5} confirmation	Rv	20	CGTGCGCACGGAAAAGCTGA
Swapping P_{ORFA6}	Fw	70	CAGCCGTTGTGGCTATATCTTTTACGCGGCGTACCAGTTAC TCTGGTCATGGTAAGCTTAATATTCCCTA
Swapping P_{ORFA6}	Rv	70	ACTTTCAAGTATATACGTCCTCAGTTTATTTCCAGACATAC TTATATAAAATCGATGAATTCGAGCTCGT
Swapping P_{ORFA6} confirmation	Rv	20	GAGATCACCTCAGTCGTCGC
Swapping P_{ORFA7}	Fw	70	CCATCCAAGAAATTGCCAAAATAGGCATCCAAAAATGACT TGTTGACCATGGTAAGCTTAATATTCCCTA
Swapping P_{ORFA7}	Rv	70	TGGTATGAATGAACTGGGCTATATTGTATCCCAAGAGCCG AAGTTGACATATCGATGAATTCGAGCTCGT
Swapping P_{ORFA7} confirmation	Rv	20	TGCCGATGTGCCCGACAGAG
Swapping P_{ORFA8}	Fw	70	TGCTTCGGTGTGAGCAGAAGCTTGATGATTCCGATCTCCA TCGCTGCGTTATCGATGAATTCGAGCTCGT
Swapping P_{ORFA8}	Rv	70	AGTTTCGATTTGTGATCAGGTAATACCTGTAAAACGGGGT CATTTTTCATGGTAAGCTTAATATTCCCTA
Swapping P_{ORFA8} confirmation	Fw	20	CCCTTGACAGATACAGCCCC
Swapping P_{ORFA9}	Fw	70	ACTTGCAAAAATTTGTCGCATCTTTTGAGCGTTTCGATGTT AAAAAGCATGGTAAGCTTAATATTCCCTA
Swapping P_{ORFA9}	Rv	70	GGAGGTAGAAGAACGGAAGACGCACCACTTGTCATCAAA TGCTCACAACAATCGATGAATTCGAGCTCGT
Swapping P_{ORFA9} confirmation	Rv	20	TCGCCCGGGGGTAGTTCTCA
Swapping P_{ORFA10}	Fw	70	TAATCCTTATAATACTGAGCCCTCGCATCCCAAAAAGCGTT CCCGTCCATGGTAAGCTTAATATTCCCTA
Swapping P_{ORFA10}	Rv	70	TTTTGAGAATGCTACAGATCAAAGCTTTAATGTTGATAGA GATAAATAGTATCGATGAATTCGAGCTCGT
Swapping P_{ORFA10} confirmation	Rv	20	CGGTCATGTCGGTGATGGCA

Swapping P_{ORFA11}	Fw	70	TTTGGATAGGCCTCAGCAGTTAACTGAGCAACAGGAGTTTGAATGGTCATGGTAAGCTTAATATTCCCTA
Swapping P_{ORFA11}	Rv	70	GATCCCCTTTCTTCAAGGACTATGGGAAAGAAAGAATGCTACAGTATTTGATCGATGAATTCGAGCTCGT
Swapping P_{ORFA11} confirmation	Rv	20	TGCACTTTGGCACCGCCTCA
Swapping P_{ORFA12}	Fw	70	CCTGCCATAGGAGCCTGGATGATTGGGTATTTCAAACTCAGACGAGTCATGGTAAGCTTAATATTCCCTA
Swapping P_{ORFA12}	Rv	70	ATCATCTTGAGTCGCACGATGCTGAAGTCAGTAATCGAAGTTTGTCTTGAATCGATGAATTCGAGCTCGT
Swapping P_{ORFA12} confirmation	Rv	20	GATCGTTGACCACGCGGCGT
Oligo dT	-	43	GCTGTCAACGATACGCTACGTAACGTTTTTTTTTTTTTTTTTT
ORFA6 qPCR	Fw	20	GCATGATTCTTTCACGGCGT
ORFA6 qPCR	Rv	20	GGCTCCTCAATAGCCGTCAT
ORFA8 qPCR	Fw	20	ATGCCACTGTTTCTGTCGGA
ORFA8 qPCR	Rv	20	GCCAATATACTGACGCCCCT
ORFA10 qPCR	Fw	20	TTTTTGGGATGCGAGGGCTC
ORFA10 qPCR	Rv	21	TCCGCCTCGTATATGTTGAGC
ORFA12 qPCR	Fw	20	ATCGGTTCAACAGGTGCAGT
ORFA12 qPCR	Rv	20	CATCGGAGACCTCTTTGGCA
ACT1 qPCR	Fw	21	GCAAACCGCTGCTCAATCTTC
ACT1 qPCR	Rv	20	TGGGGCTCTGAATCTTTCGT

Table S4. List of plasmids used and generated in this work.

Plasmid	Construct	Source
pRS426-P_{GAL1}-*Luc*	KanMxRV-P_{GAL1}-*Luc*-$CYC1_{ter}$	Salinas et al., 2018
pRS426-P_{5XGAL1}-*Luc*	KanMxRV-P_{5XGAL1}-*Luc*-$CYC1_{ter}$	Salinas et al., 2018
pRS316-FUN-$LOV^{SP\text{-}Hph}$	HphMxRV-P_{PGK1}-WC-1-*GAL4* DBD-$ADH1_{ter}$ – P_{TDH3}-VVD-*GAL4* AD-$CYC1_{ter}$	Figueroa et al., 2022
pRS316-P_{GAL1}-*sfGFP*	KanMxRV-P_{GAL1}-*sfGFP*-$CYC1_{ter}$	This work
pRS316-P_{5XGAL1}-*sfGFP*	KanMxRV-P_{5XGAL1}-*sfGFP*-$CYC1_{ter}$	This work
